# B-cell activity markers are associated with different disease activity domains in primary Sjögren’s syndrome

**DOI:** 10.1093/rheumatology/key063

**Published:** 2018-03-28

**Authors:** Katherine James, Chimwemwe Chipeta, Antony Parker, Stephen Harding, Simon J Cockell, Colin S Gillespie, Jennifer Hallinan, Francesca Barone, Simon J Bowman, Wan-Fai Ng, Benjamin A Fisher, UK Primary Sjögren’s Syndrome Registry

**Affiliations:** 1 Musculoskeletal Research Group, Institute of Cellular Medicine, Newcastle University, Newcastle, UK; 2 Interdisciplinary Computing and Complex BioSystems (ICOS) Research Group, Newcastle University, Newcastle, UK; 3 Rheumatology Research Group, Institute of Inflammation and Ageing, University of Birmingham, Birmingham, UK; 4 Department of Clinical R&D, The Binding Site Group Ltd, Edgbaston, UK; 5 Bioinformatics Support Unit, Newcastle University, Newcastle, UK; 6 School of Mathematics & Statistics, Newcastle University, Newcastle, UK; 7 Department of Biological Sciences, Macquarie University, Sydney, Australia; 8 Rheumatology Department, University Hospitals Birmingham NHS Foundation Trust, Birmingham, UK

**Keywords:** Sjögren’s syndrome, B cells, B-cell activating factor, β-2 microglobulin, free light chains, disease activity, biomarker

## Abstract

**Objectives:**

B-cell activating factor (BAFF), β-2 microglobulin (β2M) and serum free light chains (FLCs) are elevated in primary SS (pSS) and associated with disease activity. We aimed to investigate their association with the individual disease activity domains of the EULAR Sjögren’s Syndrome Disease Activity Index (ESSDAI) in a large well-characterized pSS cohort.

**Methods:**

Sera from pSS patients enrolled in the UK Primary Sjögren’s Syndrome Registry (UKPSSR) (*n* = 553) and healthy controls (*n* = 286) were analysed for FLC (κ and λ), BAFF and β2 M. Pearson correlation coefficients were calculated for patient clinical characteristics, including salivary flow, Schirmer’s test, EULAR Sjögren’s Syndrome Patient Reported Index and serum IgG levels. Poisson regression was performed to identify independent predictors of total ESSDAI and ClinESSDAI (validated ESSDAI minus the biological domain) scores and their domains.

**Results:**

Levels of BAFF, β2M and FLCs were higher in pSS patients compared to controls. All three biomarkers associated significantly with the ESSDAI and the ClinESSDAI. BAFF associated with the peripheral nervous system domain of the ESSDAI, whereas β2M and FLCs associated with the cutaneous, biological and renal domains. Multivariate analysis showed BAFF, β2M and their interaction to be independent predictors of ESSDAI/ClinESSDAI. FLCs were also shown to associate with the ESSDAI/ClinESSDAI but not independent of serum IgG.

**Conclusion:**

All biomarkers were associated with total ESSDAI scores but with differing domain associations. These findings should encourage further investigation of these biomarkers in longitudinal studies and against other disease activity measures.

**Rheumatology key messages**

- B-cell activity markers correlate with systemic disease activity in primary Sjögren’s syndrome.
- Different B-cell activity biomarkers had differing systemic disease domain associations in primary Sjögren’s syndrome.
- The role of these biomarkers should be further established in longitudinal studies in primary Sjögren’s syndrome.

## Introduction

Primary SS (pSS) is characterized by autoimmune inflammation of exocrine glands resulting in severe dryness. B-cell hyperactivity has been implicated in the pathogenesis and may contribute to the development of systemic manifestations [1, 2]. Systemic manifestations vary between patients and it is unclear how this heterogeneity is reflected in the underlying pathogenesis.

The EULAR Sjögren’s Syndrome Disease Activity Index (ESSDAI), was introduced with the aim of providing clinicians with a way of objectively measuring disease activity in pSS [3]. The ESSDAI is comprised of 12 disease activity domains, and is now the most commonly used primary outcome measure in pSS clinical trials. In order to avoid confounding in biomarker studies the ClinESSDAI was developed, in which the biological domain has been removed [4]. However, the ESSDAI has other limitations, such as its dependence on physician assessment, the difficulty of accurately assessing change in some domains, and the heavy skewing of the population towards low scores [5]. Therefore, there is interest in developing biomarkers of disease activity such as histopathology [6, 7], US [8, 9] and serum measures of B-cell activity. Proposed B-cell biomarkers include B-cell activating factor (BAFF) [10], β-2 micgroglobulin (β2M) [11, 12] and immunoglobulin free light chains (FLCs) [11]. BAFF promotes the survival and proliferation of B cells and mice overexpressing BAFF develop Sjögren’s-like features [2].

β2M forms part of the HLA class 1 molecule and enters the serum when it is shed from the cell membrane. It can be used as a marker for disease activity in conditions with high cell turnover and may act as a non-specific marker of plasma cell activity.

During immunoglobulin (Ig) synthesis, light chains are produced in excess of heavy chains. These excess light chains are secreted and are a marker of plasma cell activity [13]. FLC levels are elevated in pSS patients compared with controls and have been linked to total disease activity [11]. FLCs have a half-life of 2–6 h, making them a more dynamic marker of plasma cell activity in comparison with IgG [13].

While many studies have investigated potential biomarkers using the ESSDAI as the dependent variable, few studies have investigated a link between biomarkers and individual disease activity domains. Understanding the association of biomarkers with specific disease activity domains is important, firstly, to provide insight into the pathogenesis of the differing extra glandular manifestations, and, secondly, to avoid confounding through the expected association of B-cell activity biomarkers with the ESSDAI biological domain or through other domain-specific effects. Therefore, this study aims to investigate the relationship between the B-cell activity biomarkers BAFF, β2M and FLCs with disease activity in a large clinically well-characterized pSS cohort [5] using the ESSDAI and ClinESSDAI and their individual domains.

## Methods

### Patient recruitment and serum analysis

Serum samples for 553 pSS patients and 286 healthy controls were retrieved from the UK primary Sjögren’s syndrome registry (UKPSSR) Biobank; representing all UKPSSR subjects with available sample at the time of testing. All patients fulfilled 2002 American European Consensus Group classification criteria.

Healthy controls were recruited via two routes. First, participants were asked to voluntarily bring one to three friends of the same gender and similar age but without SS. Second, advertisements were placed at the recruiting centres. All healthy volunteers were asked to complete a general health questionnaire to screen for the presence of any significant autoimmune conditions. Only those subjects with no self-reported autoimmune conditions were included. Ethics committee approval was granted by the North West Haydock Research Ethics Committee and all subjects gave written informed consent in compliance with the Declaration of Helsinki.

Samples were analysed for serum FLCκ and FLCλ and β2M on the SPAPLUS turbidimeter (The Binding Site Group, Birmingham, UK). Combined FLC concentrations were determined by summating the individual FLCκ and FLCλ results (ΣFLC). BAFF was measured using a commercially available enzyme immunoassay (Quantikine, R&D Systems, Abingdon, UK) following the manufacturer’s instructions (serum reference interval = 584–1186 pg/ml, according to product insert).

### Data analysis

Assay data were log transformed and scaled prior to statistical analysis. Pearson correlation coefficients were calculated for patient characteristics and serum levels. Statistically significant *r* values of 0.10–0.29 were considered to be a weak association, 0.30–0.49 moderate and 0.50–1.0 strong. Poisson regression was performed to determine predictors for ESSDAI and ClinESSDAI and their subdomains, as the outcomes were discrete, with activity scores in some domains being mutually independent. A *P* < 0.05 was considered significant (after adjustment using the Benjamini-Hochberg false discovery rate where necessary). Analyses were performed using the R environment for statistical computing.

## Results

### Patient characteristics and serum biomarker levels

Five hundred and fifty-three pSS patients and 286 healthy controls were analysed. Most patients and controls were female (95.1% *vs* 90.9%) and of Caucasian ethnicity (93.7% *vs* 88.1%). However, mean age was higher in the pSS group (59.1 years; ±12.3) compared with controls (48.0 years; ±11.12).

Levels of BAFF, β2M and FLCs were higher in pSS patients compared with controls (supplementary Fig. S1, available at *Rheumatology* online). ∑FLC strongly correlated with β2M (*r* = 0.75, *P* < 0.001) but only weakly with BAFF (*r* = 0.11, *P* < 0.001). BAFF and β2M showed a moderate correlation (*r *= 0.39, *P* < 0.001).

Abnormal κ/λ FLC ratios were found in 101 (18.3%) pSS patients and 25 (8.7%) controls. All abnormal κ/λ FLC ratios were due to disproportionally high FLCκ levels. A comparison of pSS subjects with and without an abnormal κ/λ FLC ratio is shown in supplementary Table S1, available at *Rheumatology* online. The abnormal ratio pSS patients had a marginally higher rate of anti-Ro/La +ve antibodies when compared with the normal ratio pSS patients (94.1% *vs* 89.2%). The median ESSDAI score of abnormal ratio patients was similar to that of normal ratio patients (4.0 *vs* 3.0; *P* = 0.27); likewise with ClinESSDAI (4.0 *vs* 3.0; *P* = 0.38). However, subjects with an abnormal ratio had a higher prevalence of activity in the biological (69.3% *vs* 46.5%, *P* < 0.001) and renal domains (6.93% *vs* 2.2%; *P* = 0.02) although numbers in the latter domain were small. Abnormal ratio patients were also more likely to have both anti-Ro and anti-La antibodies (81.2% *vs* 70.6%, *P* = 0.04). Although an abnormal κ/λ FLC ratio suggestive of clonality could be scored as low disease activity on the biological domain [14], all scoring was undertaken without knowledge of FLC values. However, only 12 out of 88 (13.6%) patients in the abnormal ratio group who had serum electrophoresis data available had a monoclonal band, and 31/101 (30.7%) patients in the group were scored as no activity on the biological domain. All subsequent analyses included pSS subjects with an abnormal κ/λ FLC ratio, although excluding them did not substantially change our findings (data not shown).

### Serum biomarkers and clinical features

Correlation of serum biomarkers and age, disease duration, symptom duration, average Schirmer’s test, unstimulated salivary flow rate, complement C3 and C4 levels and serum IgG are shown in supplementary Table S2, available at *Rheumatology* online. FLCs showed a moderate correlation with serum IgG levels. FLCκ and ΣFLC had a weak but statistically significant inverse correlation with C4 levels. FLCs did not correlate with other clinical features. BAFF had a weak but significant correlation with disease duration (*r* = 0.11, *P* < 0.01) and symptom duration (*r* = 0.14, *P* < 0.001), and a weak inverse correlation with average Schirmer’s (*r* = −0.11, *P* < 0.05). β2M correlated weakly with age (*r* = 0.20, *P* < 0.001) and IgG levels (*r* = 0.21, *P* < 0.001), and had a weak inverse correlation with average Schirmer’s (*r* = −0.11, *P* < 0.01).

### Biomarker relationship with the ESSDAI and its domains

Complete data for ESSDAI and IgG levels were available for 539 pSS subjects. All exploratory biomarkers were found to have a statistically significant positive association with both total ESSDAI and ClinESSDAI scores (*P* < 0.001 for all) (supplementary Table S3, available at *Rheumatology* online).

**Fig. 1:**
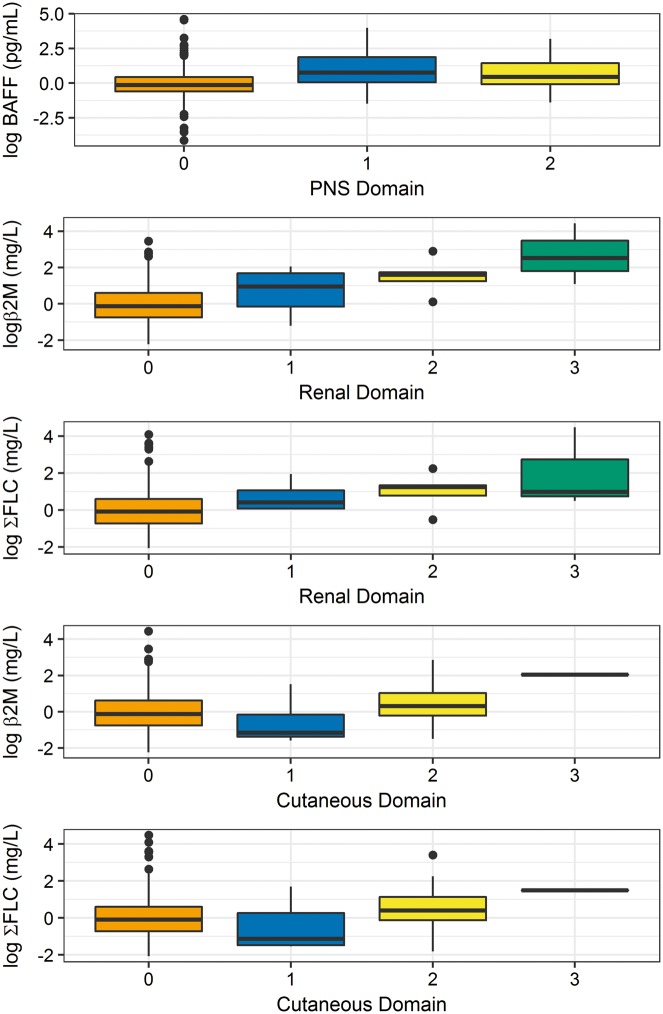
Relationship of serum biomarkers with activity levels of their associated domains (biological domain excluded)

Table 1 shows the results of Poisson regression of serum biomarkers against the ESSDAI domains. BAFF showed a statistically significant association with the peripheral nervous system (PNS) domain (*P* < 0.001), but not with any other domain. β2M and FLCs were significantly associated with the cutaneous, biological and renal domains (*P* < 0.001 for all). Figure 1 shows the level of serum biomarkers in relation to the activity levels of their associated domains (biological domain excluded).

**Table 1:** Poisson regression *P-*values for association of biomarkers with individual ESSDAI domains

ESSDAI domain	BAFF	β2M	FLCλ	FLCκ	ΣFLC	IgG
Constitutional	2.93E-01	2.13E-01	2.93E-01	2.13E-01	2.13E-01	2.13E-01
Lymphadenopathy	6.71E-01	6.71E-01	6.29E-01	1.94E-01	1.94E-01	7.80E-01
Glandular	8.35E-01	4.35E-01	3.91E-01	1.74E-01	1.74E-01	1.74E-01
Articular	4.03E-01	7.73E-01	4.03E-01	4.03E-01	4.03E-01	9.14E-02
Cutaneous	2.15E-01	6.90E-04***	1.14E-03***	9.56E-06***	4.21E-05***	4.93E-03**
Respiratory	1.62E-01	1.62E-01	9.33E-01	9.33E-01	9.33E-01	3.07E-01
Muscular	3.01E-01	7.99E-02	7.06E-01	3.01E-01	3.53E-01	4.14E-01
Peripheral nervous system	2.25E-05***	5.22E-02	8.90E-01	8.90E-01	8.90E-01	8.90E-01
Haematological	1.73E-01	1.73E-01	5.91E-01	4.28E-01	4.50E-01	5.91E-01
Biological	5.97E-01	2.94E-15***	5.03E-29***	1.32E-35***	9.84E-36***	2.00E-49***
Renal	1.57E-01	3.71E-16***	1.43E-09***	1.01E-10***	8.38E-11***	7.07E-02

CNS domain excluded since only two patients active.

** *P *< 0.01.

*** *P *< 0.001. BAFF: B cell activating factor; β2M: β-2 microglobulin; FLC: free light chain.

The association of FLC and β2M with the total ESSDAI score remained after correction for estimated glomerular filtration rate (eGFR), (supplementary Table S3, available at *Rheumatology* online). Furthermore, both FLC and β2M remain associated with the renal domain after correction for eGFR (data not shown).

### Multivariate ESSDAI

On multivariate analysis of serum biomarkers against the total ESSDAI (supplementary Table S4, available at *Rheumatology* online), BAFF and β2M were found to be independent predictors of the ESSDAI. They were also found to have an interaction. FLCs offered no additional value to measured IgG on multivariate regression. Broadly similar results were seen in multivariate analysis of biomarkers in relation to total ClinESSDAI scores. β2M, ∑FLC and the interaction of BAFF and β2M were all found to be independent predictors within the same model. However, the overall ability of these models to explain variation in actual ESSDAI or CLinESSDAI scores was small.

## Discussion

Serum FLCs, BAFF and β2M levels all showed statistically significant associations with total ESSDAI/ClinESSDAI scores, with BAFF and β2M showing both independent and multiplicative associations on multivariate testing. BAFF was significantly associated with the PNS and no other domains whereas β2M and all FLCs were significantly associated with the cutaneous, biological, and renal domains. These differing associations are consistent with there being only weak to moderate correlation of FLCs and β2M with BAFF levels, and indicate that these biomarkers provide complementary and non-redundant information.

Previous studies have reported an association of FLCs and β2M with the total ESSDAI score [11, 12, 15], but did not explore relationships with the individual domains. The relationship between serum BAFF and ESSDAI domains has been explored in two small studies [10, 16]. BAFF was associated with lymphadenopathy, glandular and pulmonary domains in a cohort of 58 patients [10], while in another study of 76 patients BAFF was associated with the constitutional, lymphadenopathy, glandular, and biological domains [16]. The latter study included a high proportion of pSS patients with lymphoproliferative disorders. These findings are in contrast to our much larger study in which BAFF only showed an association with the PNS domain. There are diverse manifestations of PNS involvement in pSS with both vasculitic and non-vasculitic neuropathies. However, BAFF is elevated in non-pSS patients with chronic inflammatory demyelinating polyneuropathy [17, 18] and is also expressed by perivascular and intramural lymphocytes in patients with non-systemic vasculitic neuropathy [19].

FLCs are excreted by the kidneys and β2M is freely filtered by the glomeruli and reabsorbed into the proximal renal tubules, where it is then degraded. Thus, renal impairment can increase serum FLCs and β2M. However, the association of these biomarkers with the renal domain remained following correction for eGFR, suggesting that this association is not an artefact introduced by renal impairment. The commonest manifestation of renal involvement in pSS is distal renal tubular acidosis due to tubulointerstitial nephritis in which infiltration of B cells and plasma cells is common [20].

Close to 20% of pSS patients were found to have an abnormal κ/λ ratio, all due to elevated κFLCs. We hypothesize that these abnormal ratios reflect the favouring of κ light chain usage by dominant public clonotypes of pSS-related autoantibodies [21].

In this study, we used ESSDAI as the gold standard for assessing disease activity. While the ESSDAI is a useful tool, it does have some limitations, and does not assess glandular activity unaccompanied by palpable swelling. Given these limitations, the inability of our multiparameter models to accurately predict ESSDAI scores does not negate the potential value of these biomarkers. While we could not identify an independent predictive value of FLCs over the more commonly measured IgG levels, the cross-sectional nature of our study means we cannot exclude their utility in longitudinal cohorts or interventional trials. The considerably shorter half-life of FLCs in comparison to IgG could allow more dynamic monitoring of B-cell hyperactivity in such settings. A key strength of our study is it being the largest to date, giving it the ability to assess the relationship of these B-cell biomarkers with ESSDAI domains. However, larger numbers still would be required for some of the domains where disease activity is rare, such as the CNS and muscular. Furthermore, the clinical significance of our findings is unclear, given the relatively weak associations observed.

In conclusion, all three biomarkers were shown to associate significantly with total ESSDAI scores but with differing domain associations. Further investigation of these biomarkers is warranted.

## Supplementary Material

Supplementary Data
