# Supplementary material for: B-cell activity markers are associated with different disease activity domains in primary Sjögren’s syndrome
Source: Rheumatology (Oxford). 2018 Mar 28;57(7):1222–7. doi: 10.1093/rheumatology/key063 (PMC6014143; doi:10.1093/rheumatology/key063)
Supplement: Supplementary Data [file key063_suppl_data.docx]

# SUPPLEMENTARY DATA

**Supplementary Table S1. Patient clinical profiles for the normal and abnormal ratio and all groups**

|  | **Normal ratio (n=452)** | **Abnormal ratio (n=101)** | **All (n=553)** |
| --- | --- | --- | --- |
| **Age at recruitment,** mean (SD) | 58.76 (12.37) | 60.75 (11.81) | 59.13 (12.28) |
| **Female** | 429 (94.9) | 97 (96.1) | 526 (95.1) |
| **Ethnicity,** Caucasian | 424 (93.8) | 94 (93.1) | 518 (93.7) |
| **Disease duration** |  |  |  |
| Years**,** mean (SD) | 7.01 (6.17) (n=449) | 7.17 (5.95) | 7.04 (6.13) (n=550) |
| **Symptom duration** |  |  |  |
| Years, mean (SD) | 12.96 (9.90) (n=450) | 14.14 (11.82) | 13.17 (10.28) (n=551) |
| **Anti Ro & AntiLa +ve** | 319 (70.58) | 82 (81.19) | 401 (72.51) |
| **Anti Ro +ve** | 402 (88.94) | 95 (94.06) | 497 (89.87) |
| **Anti La +ve** | 323 (71.46) | 82 (81.19) | 405 (73.24) |
| **ESSDAI**, median (IQR) | 3.00 (1.00-7.00) (n=451) | 4.00 (2.00-8.00) | 4.00 (1.00-7.00) (n=552) |
| **ClinESSDAI**, median (IQR) | 3.00 (0.00-7.00) (n=451) | 4.00 (0.00-8.00) | 4.00 (0.00-7.25) (n=552) |
| Constitutional > 0 | 108 (23.9) | 21 (20.8) | 129 (23.3) |
| Lymphadenopathy > 0 | 19 (4.2) | 8 (7.9) | 27 (4.88) |
| Glandular > 0 | 79 (17.5) | 20 (19.8) | 99 (17.90) |
| Articular > 0 | 139 (30.8) | 31 (30.7) | 170 (30.74) |
| Cutaneous > 0 | 33 (7.3) | 10 (9.9) | 43 (7.78) |
| Respiratory > 0 | 41 (9.1) (n=451) | 10 (9.9) | 51 (9.24) (n=451) |
| Muscular > 0 | 11 (2.4) | - | 11 (1.98) |
| PNS > 0 | 20 (4.4) | 5 (5.0) | 25 (4.52) |
| CNS > 0 | 2 (0.44) | - | 2 (0.36) |
| Hematological > 0 | 77 (17.0) | 21 (20.8) | 98 (17.18) |
| Biological > 0 | 210 (46.5) | 70 (69.3) | 280 (50.63) |
| Renal > 0 | 10 (2.2) | 7 (6.93) | 17 (3.07) |
| **ESSPRI**, median (IQR) | 5.67 (4.00-7.00) (n=447) | 5.33 (3.67-6.67) | 5.67 (3.67-7.00) (n=448) |
| Fatigue | 6.00 (4.00-8.00) (n=447) | 5.00 (3.00-7.00) | 6.00 (3.75-8.00) (n=448) |
| Dryness | 7.00 (4.00-8.00) (n=447) | 6.00 (4.00-8.00) | 6.00 (4.00-8.00) (n=448) |
| Pain | 5.00 (2.00-7.00) (n=447) | 4.00 (2.00-7.00) | 5.00 (2.00-7.00) (n=448) |
| **SSDDI**, median (IQR) | 4.00 (2.00-5.00) | 4.00 (3.00-5.00) | 4.00 (2.00-5.00) |

Values given as n (%) unless otherwise stated. PNS: peripheral nervous system; CNS: central nervous system; ESSDAI: European league against rheumatism Sjögren’s syndrome disease activity index; ClinESSDAI: ESSDAI minus the biological domain; ESSPRI: European League Against Rheumatism Sjögren’s syndrome patient reported index; SSDDI: Sjögren’s syndrome disease damage index

**Supplementary Table S2. Correlations between BAFF, B2M, IgG, FLC and other clinical factors**

| **Factor** | **BAFF** | **β2M** | **FLCκ** | **FLCλ** | **ΣFLC** | **IgG** |
| --- | --- | --- | --- | --- | --- | --- |
| **Age** | 0.097* | 0.199*** | 0.004 | -0.047 | -0.013 | -0.163*** |
| **Disease duration** (n=550) | 0.114** | 0.010. | -0.061 | -0.039 | -0.057 | -0.100* |
| **Symptom duration** (n=551) | 0.143*** | 0.035 | -0.060 | -0.068 | -0.067 | -0.115** |
| **Average Schirmer’s** (n=540) | -0.105* | -0.112** | -0.033 | -0.055 | -0.043 | -0.066 |
| **Saliva flow** | -0.069 | -0.095* | -0.068 | -0.053 | -0.068 | -0.022 |
| **C3** (n=535) | 0.098* | -0.009 | -0.032 | -0.029 | -0.033 | -0.017 |
| **C4** (n=535) | -0.049 | -0.090* | -0.137** | -0.092* | -0.130** | -0.045 |
| **IgG** (n=542) | -0.063 | 0.211*** | 0.309*** | 0.359*** | 0.349*** | - |

Patients with with abnormal FLC kappa/lambda ratio are included. **p* < 0.05, ***p* < 0.01, ****p* < 0.001. BAFF: B cell activating factor; β2M: β-2 microglobulin; FLC: free light chain; C3: compliment component 3; C4: compliment component 4.

**Supplementary Table S3. Poisson regression of ESSDAI**

|  | **ESSDAI** |  |  | **ClinESSDAI** |  |  |
| --- | --- | --- | --- | --- | --- | --- |
|  | ***p*-value** | **OR** | **95% CI** | ***p*-value** | **OR** | **95% CI** |
| **BAFF** | 1.69E-20*** | 1.184 | 1.142-1.226 | 3.21E-24*** | 1.201 | 1.159-1.243 |
| **β2M** | 1.22E-41*** | 1.278 | 1.234-1.323 | 9.32E-30*** | 1.231 | 1.188-1.275 |
| **FLCλ** | 6.79E-19*** | 1.184 | 1.141-1.229 | 3.80E-06*** | 1.094 | 1.054-1.136 |
| **FLCκ** | 4.34E-26*** | 1.214 | 1.171-1.257 | 1.43E-08*** | 1.115 | 1.075-1.157 |
| **ΣFLC** | 1.26E-25*** | 1.214 | 1.171-1.258 | 1.62E-08*** | 1.115 | 1.074-1.156 |
| **IgG** | 4.57E-05*** | 1.081 | 1.041-1.122 | 1.87E-02* | 0.955 | 0.920-0.992 |
| **β2M^a^** | 3.50E-34*** | 1.267 | 1.220-1.315 | 3.00E-22*** | 1.211 | 1.166-1.258 |
| **FLCλ^a^** | 2.43E-15*** | 1.166 | 1.122-1.210 | 3.66E-04*** | 1.073 | 1.033-1.114 |
| **FLCκ^a^** | 7.87E-21*** | 1.194 | 1.150-1.238 | 1.38E-05*** | 1.090 | 1.050-1.131 |
| **ΣFLC^a^** | 1.45E-20*** | 1.194 | 1.150-1.239 | 1.38E-05*** | 1.090 | 1.049-1.131 |

^a^With eGFR. **p* < 0.05, ***p* < 0.01, ****p* < 0.001. BAFF: B cell activating factor; β2M: β-2 microglobulin; FLC: free light chain; ESSDAI: European League Against Rheumatism Sjögren’s syndrome disease activity index; ClinESSDAI: ESSDAI minus the biological domain.

**Supplementary Table S4. Multivariate regression**

| **Model** | **Factor** | **ESSDAI** | **ClinESSDAI** |
| --- | --- | --- | --- |
| BAFF+β2M+IgG | BAFF | 1.94E-05*** | 9.57E-06*** |
|  | β2M | 2.27E-22*** | 1.45E-23*** |
|  | IgG | 9.16E-01 | 3.03E-09*** |
| BAFF+β2M + ΣFLC | BAFF | 8.46E-07*** | 2.23E-07*** |
|  | β2M | 1.87E-07*** | 1.64E-11*** |
|  | cFLC | 2.86E-02* | 5.46E-02 |
| BAFF*β2M + ΣFLC | BAFF | 1.02E-01 | 1.07E-01 |
|  | β2M | 7.18E-06*** | 5.75E-09*** |
|  | ΣFLC | 6.76E-03** | 1.88E-01 |
|  | BAFF: β2M | 2.18E-12*** | 2.70E-15*** |
| BAFF*β2M + ΣFLC | BAFF | 1.02E-01 | 1.07E-01 |
|  | β2M | 7.18E-06*** | 5.75E-09*** |
|  | cFLC | 6.76E-03** | 1.88E-01 |
|  | BAFF: β2M | 2.18E-12*** | 2.70E-15*** |

BAFF: B cell activating factor; β2M: β-2 microglobulin; FLC: free light chain; ESSDAI: European League Against Rheumatism Sjögren’s syndrome disease activity index; ClinESSDAI: ESSDAI minus the biological domain.

**Figure 1. Serum levels of B cell biomarkers in pSS patients and healthy controls**


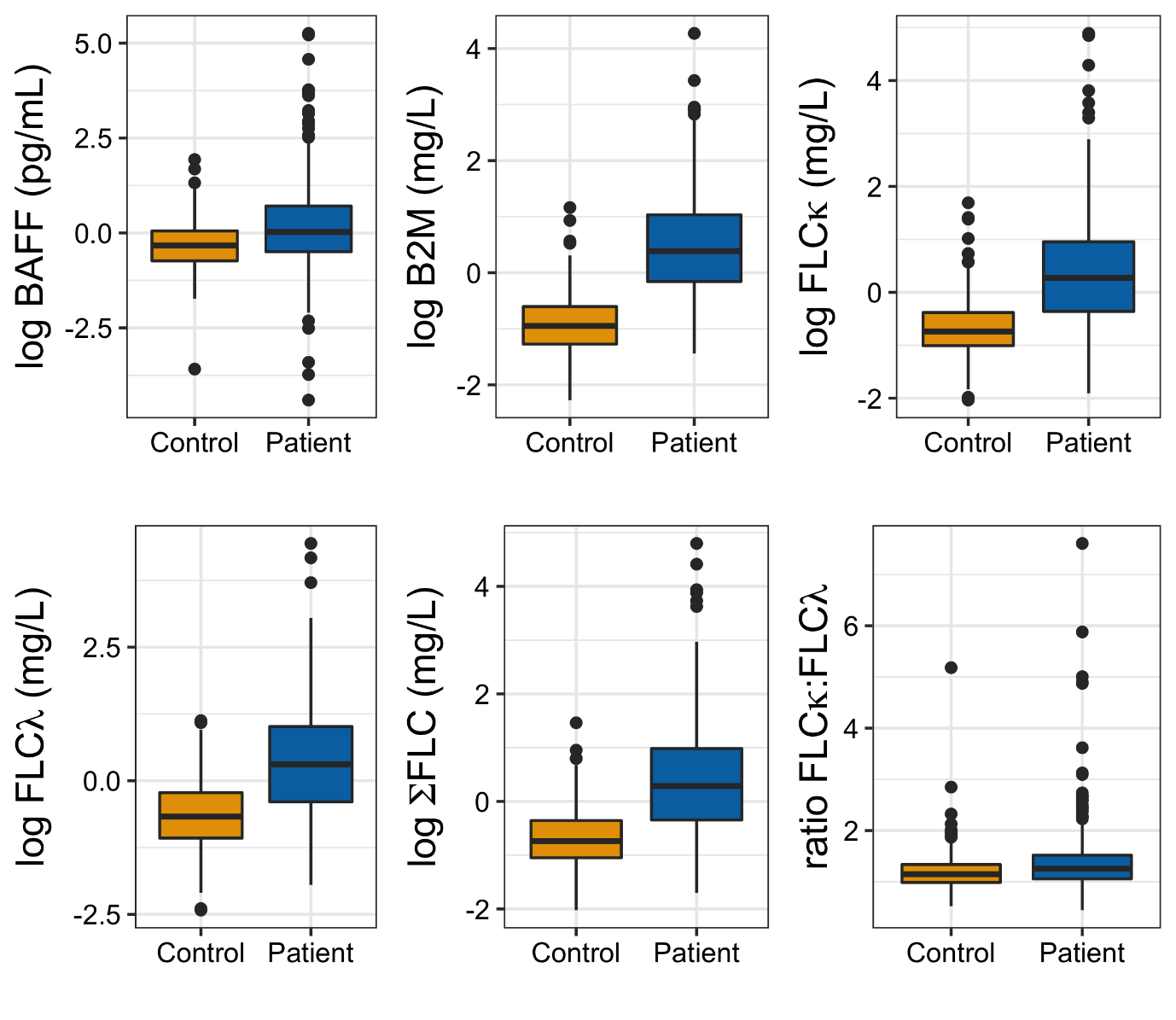


P values for serum BAFF, B2M, FLCκ, FLCλ, ƩFLC levels and FLC κ/λ ratio in pSS versus controls are 5.20E-12, 4.13E-103, 6.18E-52, 1.29E-43, 1.30E-52, 8.06E-06, respectively. BAFF: B cell activating factor; B2M: β-2 microglobulin; FLC: free light chain; pSS: primary Sjögren’s syndrome.
